# Nrf2/Keap1 system regulates vascular smooth muscle cell apoptosis for vascular homeostasis: role in neointimal formation after vascular injury

**DOI:** 10.1038/srep26291

**Published:** 2016-05-20

**Authors:** Takashi Ashino, Masayuki Yamamoto, Satoshi Numazawa

**Affiliations:** 1 Division of Toxicology, Department of Pharmacology, Toxicology & Therapeutics, Showa University School of Pharmacy, 1-5-8 Hatanodai, Shinagawa-ku, Tokyo 142-8555, Japan; 2 Department of Medical Biochemistry, Tohoku University Graduate School of Medicine, 2-1 Seiryo-cho, Aoba-ku, Sendai 980-8575, Japan

## Abstract

Abnormal increases in vascular smooth muscle cells (VSMCs) in the intimal region after a vascular injury is a key event in developing neointimal hyperplasia. To maintain vascular function, proliferation and apoptosis of VSMCs is tightly controlled during vascular remodeling. NF-E2-related factor 2 (Nrf2)/Kelch-like ECH-associated protein 1 (Keap1) system, a key component of the oxidative stress response that acts in maintaining homeostasis, plays an important role in neointimal hyperplasia after a vascular injury; however, the role of Nrf2/Keap1 in VSMC apoptosis has not been clarified. Here we report that 14 days after arterial injury in mice, TUNEL-positive VSMCs are detected in both the neointimal and medial layers. These layers contain cells expressing high levels of Nrf2 but low Keap1 expression. In VSMCs, Keap1 depletion induces features of apoptosis, such as positive TUNEL staining and annexin V binding. These changes are associated with an increased expression of nuclear Nrf2. Simultaneous Nrf2 depletion inhibits Keap1 depletion-induced apoptosis. At 14 days after the vascular injury, Nrf2-deficient mice demonstrated fewer TUNEL-positive cells and increased neointimal formation in the neointimal and medial areas. The results suggest that the Nrf2/Keap1 system regulates VSMC apoptosis during neointimal formation, thereby inhibiting neointimal hyperplasia after a vascular injury.

Vascular smooth muscle cells (VSMCs) play a crucial role in vascular wound repair after injury^1^,^2^. Under normal conditions, VSMCs exhibit a contractile phenotype and a low turnover rate. After a vascular injury, the intima is disrupted, triggering VSMCs to convert to a synthetic phenotype, thereby accelerating VSMC proliferation and migration and causing vessel remodeling^2^. In contrast, numerous studies have demonstrated that VSMC apoptosis is also increased in atherosclerotic lesions after arterial injury^3^,^4^. Abnormal growth of VSMCs in the intimal layer of blood vessels in response to injury causes neointimal hyperplasia, a key event in the development of arteriosclerosis and restenosis^2^. Therefore, during vascular wound healing, balanced control of VSMC growth and death critically regulates the composition of the healed vessel wall and luminal patency.

Reactive oxygen species (ROS) have been recognized as intracellular signaling molecules that are involved in physiological repair processes after a vascular injury^5^,^6^. In VSMCs, physiological levels of ROS produced by stimulating growth factors, such as platelet-derived growth factor (PDGF), serve to regulate many growth and migratory responses, thereby contributing to wound repair^6^,^7^. Conversely, excessive and/or persistent stimulation of VSMCs by ROS inflicts oxidative stress and can alter the functions of VSMCs, enhance proliferation and migration, and induce resistance to apoptosis. These changes can contribute to the development of cardiovascular diseases, including arteriosclerosis and myocardial hypertrophy^8^,^9^. Several studies have reported that ROS generation is increased during restenosis after angioplasty^10^,^11^. Moreover, in arteriosclerotic lesions, the activation of NAD(P)H oxidase, a major source of ROS in the vessel wall, was observed^7^,^12^. In addition, risk factors for the development of arteriosclerosis, e.g., diabetes and hypertension, increase ROS levels in the vasculature^7^. Thus, cellular defenses against oxidative stress are critical for maintaining vascular homeostasis.

To prevent ROS-induced irreversible tissue damage, redox status is tightly controlled by various antioxidant systems. The nuclear factor erythroid 2-related factor 2 (Nrf2) and its suppressor Kelch-like ECH-associated protein 1 (Keap1) system plays a critical role in cellular resistance to oxidative stress^13^. Under normal conditions, Nrf2 is rapidly trapped by Keap1 in the cytoplasm, promoting ubiquitin-dependent proteolysis. However, in the presence of ROS, Keap1 is inactivated via the modification of cysteine residues. Therefore, Nrf2 is stabilized and translocated to the nucleus. Nrf2 heterodimerizes with small Maf proteins and activates the expression of target genes, such as NAD(P)H: quinone oxidoreductase-1 (Nqo1) and heme oxygenase-1 (Hmox1), by binding to the antioxidant responsive element^13^,^14^. Thus, the Nrf2/Keap1 pathway is assembled as a cytoprotective system to sensitively and quickly respond to oxidative stress. The Nrf2/Keap1-mediated protection against oxidative stress has been observed in VSMCs. Knockdown of Nrf2 prolongs the elevation of intracellular ROS levels in response to PDGF stimulation and enhances PDGF-induced VSMC migration^15^. Accordingly, Nrf2 and its dependent genes Hmox1 and Nqo1 inhibit VSMC proliferation and migration following exposure to PDGF or serum^16^,^17^,^18^. In addition, Nrf2 gene-depletion increases neointimal formation in response to transluminal mechanical injury of the femoral artery in a mouse model of arteriosclerosis^15^. These reports indicate that Nrf2 functions as a key regulator of vascular homeostasis and protects against arteriosclerosis and subsequent vascular occlusive diseases. However, the direct involvement of Nrf2 in VSMC apoptosis during physiological vascular remodeling after injury has not yet been demonstrated.

In this study, we demonstrated that terminal deoxynucleotidyl transferase-mediated dUTP nick-end labeling (TUNEL)-positive cells were detected in both the neointimal and medial layers after the vascular injury. In these layers, smooth muscle cell-specific α-actin (αSMA)-positive cells and cells expressing high Nrf2 levels were also detected. The Keap1 levels were decreased in the injured vessels. In cultured VSMCs, siRNA-mediated depletion of Keap1 increased morphological features of apoptosis, annexin V binding, and TUNEL staining. These effects were ameliorated by co-knockdown with Nrf2. In Nrf2-deficient^(−/−)^ mice, fewer TUNEL-positive cells are observed in the neointimal and medial layers, and increased neointimal formation is observed after the vascular injury. These findings suggest that the Nrf2/Keap1 system regulates VSMC apoptosis during vascular remodeling, thereby counterbalancing the effects of proliferation, which may reduce neointimal hyperplasia after a vascular injury.

## Results

### Increased expression of Nrf2 associated with a decrease in Keap1 and VSMC apoptosis during the middle stages of neointimal expansion in response to vascular injury

**Figure 1:**
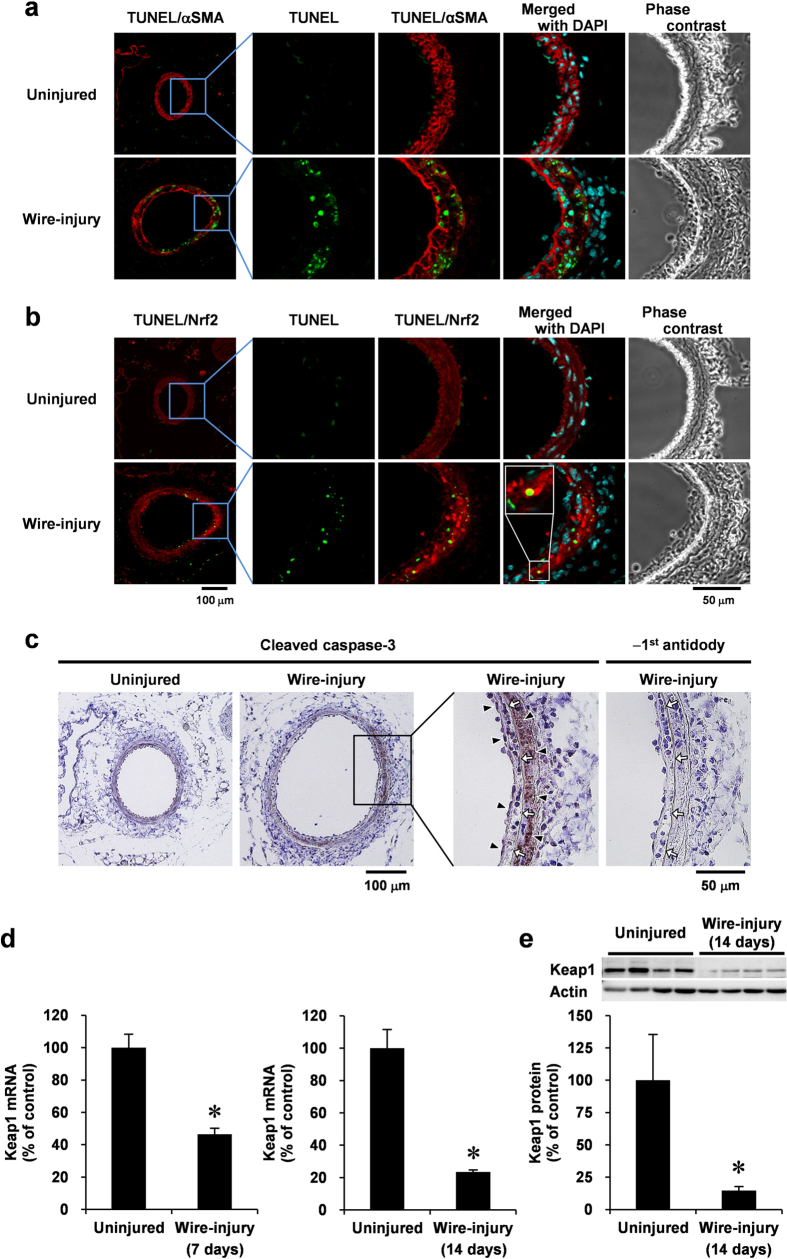
VSMC apoptosis in the middle stages of neointimal expansion after the vascular injury is associated with a high expression of Nrf2, decrease in Keap1, and caspase-3 activation. Femoral arteries uninjured or injured (14 days later) co-stained with TUNEL (green), DAPI (blue), and anti-αSMA (red; (**a**)) or anti-Nrf2 (red; (**b**)) antibodies for immunofluorescence analysis, or stained with anti-cleaved caspase-3 antibody (**c**) for immunohistochemical analysis. Fluorescence images were taken by confocal microscopy under fixed exposure conditions. Arrowheads indicate cleaved caspase-3; white arrows indicate internal elastic lamina. Keap1 mRNA (**d**) and protein (**e**) levels of uninjured or injured (7 or 14 days later) femoral artery were analyzed using real-time PCR and Western blotting, respectively. Data are expressed as mean ± SEM of four vessels. *P < 0.05 vs. uninjured vessels.

To examine the distribution of apoptotic cells and Nrf2 expression in the middle stages of neointimal expansion (VSMC proliferative phase) after the vascular injury *in vivo*, we injured the femoral arteries of mice and performed TUNEL analysis and immunofluorescence staining with antibodies against Nrf2 or αSMA, a VSMC marker. TUNEL-positive cells were not observed and Nrf2 was weakly expressed in uninjured vessels (Fig. 1a,b). Fourteen days after the injury, TUNEL-positive cells were detected in both the developing neointimal and medial layers, which are the two layers composed of αSMA-positive cells (Fig. 1a). Moreover, a large number of cells expressing high Nrf2 levels were observed in the areas where apoptosis was detected (Fig. 1b). Apoptosis is initiated by the activation of caspase-3, which requires proteolytic processing of inactive zymogen. Therefore, we examined the generation of cleaved caspase-3 in wire-injured vessels. Immunohistochemical analyses revealed that activated caspase-3 levels were increased in the neointimal and medial layers, and the distribution of activated caspase-3 overlaps with areas of high Nrf2-expression (Fig. 1c).

Keap1 is a known negative regulator of Nrf2 by mediating its proteasomal degradation^13^. Therefore, we examined the influence of the vascular injury on Keap1 expression. At 7 days after injury, vascular levels of Keap1 mRNA were decreased in the injured vessels from the early stages of neointimal expansion. At 14 days, the decreased levels of Keap1 mRNA were observed in the injured vessels (Fig. 1d). Consistent with mRNA levels, Keap1 protein was also significantly decreased in the injured vessels at 14 days after injury (Fig. 1e).

### Keap1 depletion induces VSMC apoptosis *in vitro*

**Figure 2:**
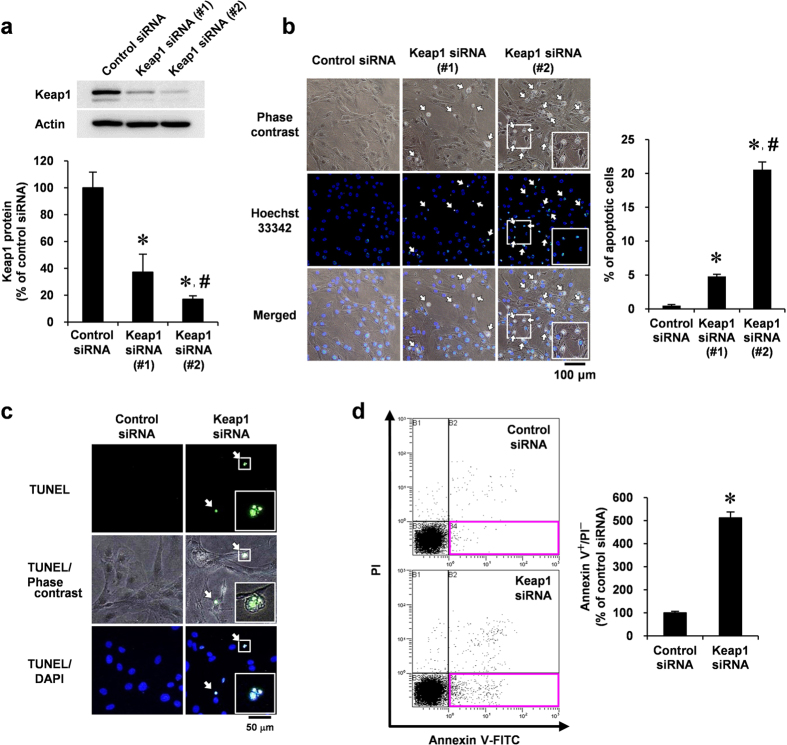
Effect of Keap1 depletion in VSMC apoptosis. (**a–d**) RASMCs were transiently transfected with Keap1 (#1 or #2) or control siRNA for 48 h. (**a**) Keap1 protein levels were analyzed by Western blotting. (**b**) Growth-arrested RASMCs were stained with Hoechst 33342 (blue). Apoptotic RASMCs exhibited shrunken morphology and fragmented nuclei with bright nuclear fluorescence indicated by arrows. The percentage of apoptotic cells were determined by counting shrunken cells in three separate view fields. (**c**) Growth-arrested RASMCs were co-stained with TUNEL (green) and DAPI (blue). Arrows indicate TUNEL positive cells. Results are representative of three independent replicates of immunofluorescence images. (**d**) Growth-arrested RASMCs were co-stained with annexin V–FITC and PI, and analyzed by flow cytometry. Apoptotic cells were defined by annexin V positive and PI negative cells (red square at right lower quadrants). Data are expressed as mean ± SEM of three independent experiments. *P < 0.05 vs. control siRNA. ^#^*P* < 0.05 vs. Keap1 siRNA (#1).

To determine the role of Keap1 depletion in VSMCs, we examined whether Keap1 depletion by siRNA could induce VSMC apoptosis using cultured rat aortic smooth muscle cells (RASMCs). The transfection of VSMCs with two kinds of Keap1 siRNAs significantly reduced Keap1 protein in whole-cell lysates (#1, 63%; #2, 83% reduction) (Fig. 2a). Keap1 depletion induced features of VSMC apoptosis, including shrunken morphology and bright nuclear fluorescence by Hoechst 33342 staining (Fig. 2b). A more effective Keap1 siRNA (#2) induced apoptosis in a greater fraction of cells (20.6% of the total cell number) than a weaker Keap1 siRNA (#1; 4.8% of the total cell number; Fig. 2b). Therefore, we used the more effective Keap1 siRNA (#2) in further experiments.

DNA fragments with 3′-OH ends are produced by the activation of endonucleases in cells undergoing apoptosis; thus, we used the TUNEL assay to detect apoptotic cells. The cells that appeared shrunken following transfection with Keap1 siRNA also had condensed and fragmented nuclei and positive TUNEL staining (Fig. 2c). Keap1 depletion-induced VSMC apoptosis was further confirmed by assessing the surface expression of phosphatidylserine using annexin V in conjunction with propidium iodide (PI), which was detected by flow cytometry. Keap1 depletion increased the number of apoptotic cells (annexin V positive and PI negative; Fig. 2d).

### Keap1 depletion induces Nrf2 stabilization followed by nuclear accumulation and target gene expression in VSMCs

**Figure 3:**
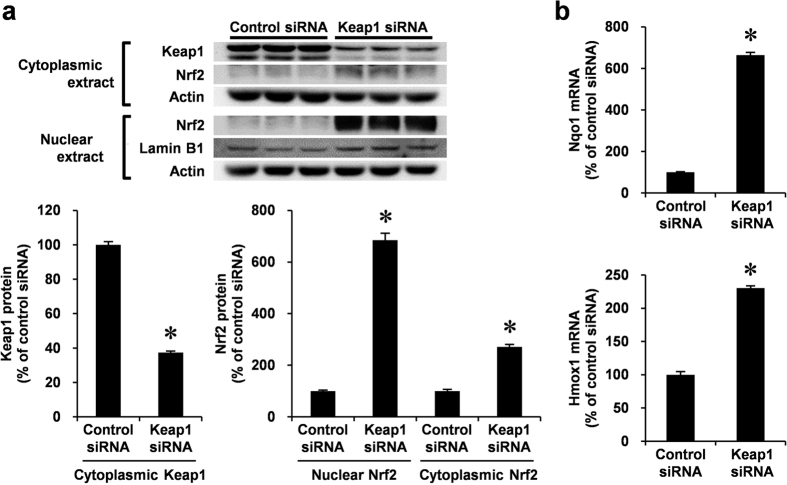
Endogenous Keap1 knockdown by siRNA and expression of Nrf2 and its target genes, including Nqo1 and Hmox1 in VSMCs. (**a,b**) RASMCs were transiently transfected with Keap1 or control siRNA for 48 h. (**a**) Nuclear and cytoplasmic protein levels were analyzed by Western blotting. Cytoplasmic and nuclear proteins were semi-quantified by normalizing with actin and lamin B protein, respectively. (**b**) mRNA levels were analyzed by real-time PCR. Data are expressed as mean ± SEM of three independent experiments. *P < 0.05 vs. control siRNA.

Functional disorder of Keap1 inhibits Nrf2 degradation^19^. Therefore, we next examined the effects of Keap1 siRNA in Nrf2 stability and subcellular localization. Keap1 siRNA significantly reduced cytoplasmic levels of Keap1. In contrast, cellular Nrf2 levels were robustly increased, particularly in the nucleus (Fig. 3a). Furthermore, Keap1 depletion induced the expression of exemplary Nrf2 target genes, including Nqo1 and Hmox1, in VSMCs (Fig. 3b).

### Simultaneous depletion of Nrf2 rescues VSMC from Keap1 depletion-induced apoptosis

**Figure 4:**
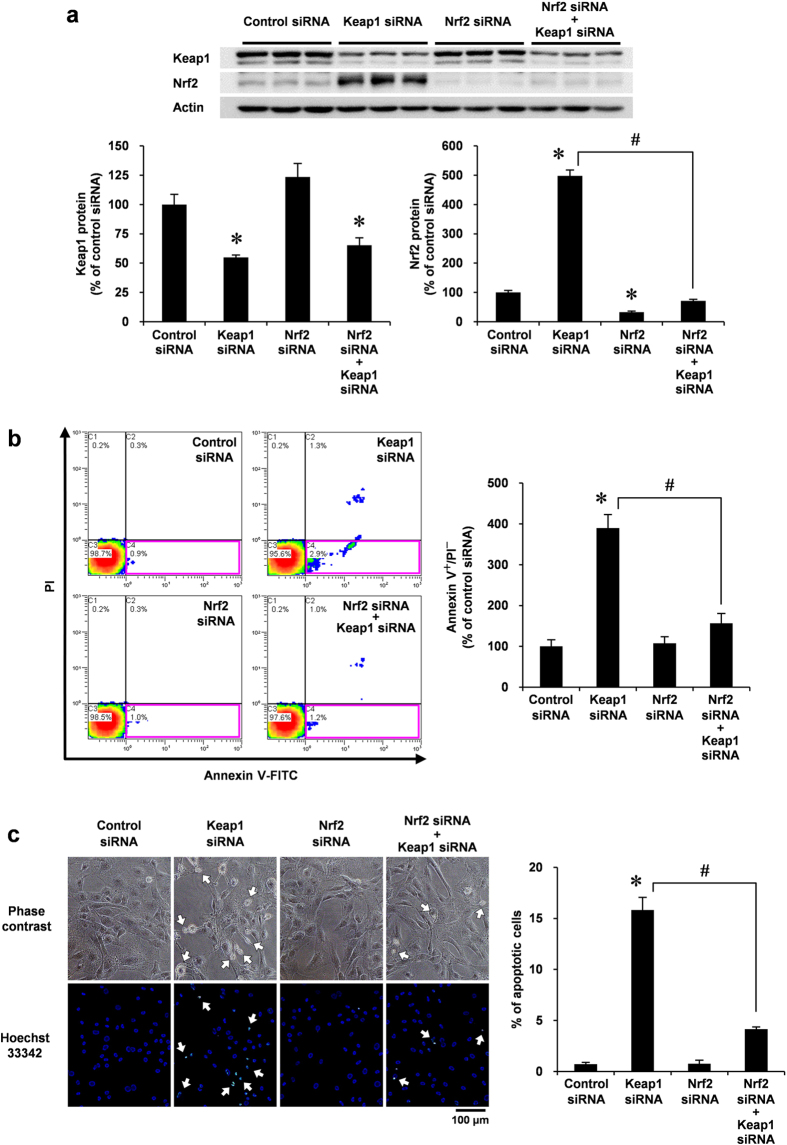
Effect of Nrf2 siRNA co-transfected with Keap1 siRNA in VSMC apoptosis. (**a–c**) RASMCs were transiently transfected with Nrf2 or control siRNA. After 24 h, the RASMCs were additively transfected with Keap1 or control siRNA for 48 h. (**a**) Protein levels in the whole cell lysate were analyzed by Western blotting. (**b**) Growth-arrested RASMCs were co-stained with annexin V–FITC and PI and were analyzed by flow cytometry. Apoptotic cells were defined as described for Fig. 2d. (**c**) Growth-arrested RASMCs were stained with Hoechst 33342 (blue). The percentage of apoptotic cells were determined as described for Fig. 2b. Arrows indicate apoptotic cells. Data are expressed as mean ± SEM of three independent experiments. *P < 0.05 vs. control siRNA. ^#^*P* < 0.05 vs. Keap1 siRNA alone.

Because Keap1 depletion increased cellular Nrf2 levels, we examined whether the overexpression of Nrf2 was involved in VSMC apoptosis. Similar to the results presented in Fig. 3a, knockdown of Keap1 increased cellular Nrf2 levels, whereas co-knockdown of Nrf2 completely ameliorated this effect in VSMCs (Fig. 4a). To investigate the role of Nrf2 in Keap1-depleted VSMCs, we next assessed annexin V/PI staining by flow cytometry, following knockdown of Keap1 and/or Nrf2. Annexin V staining of Nrf2-knockdown cells did not significantly differ from that of control cells. In Keap1-depleted cells, the number of cells undergoing apoptosis (annexin V positive but PI negative) was reduced by co-knockdown of Nrf2 (Fig. 4b). Corresponding with flow cytometry analysis, the morphological characteristics of apoptosis that were detected by Hoechst 33342 staining also revealed that the simultaneous knockdown of Nrf2 significantly inhibited Keap1 depletion-induced morphological changes in VSMCs (Fig. 4c). These results suggest that Nrf2 plays a role in Keap1 depletion-induced VSMC apoptosis.

### Nrf2/Keap1 system is involved in caspase-3/7 activation in VSMCs

**Figure 5:**
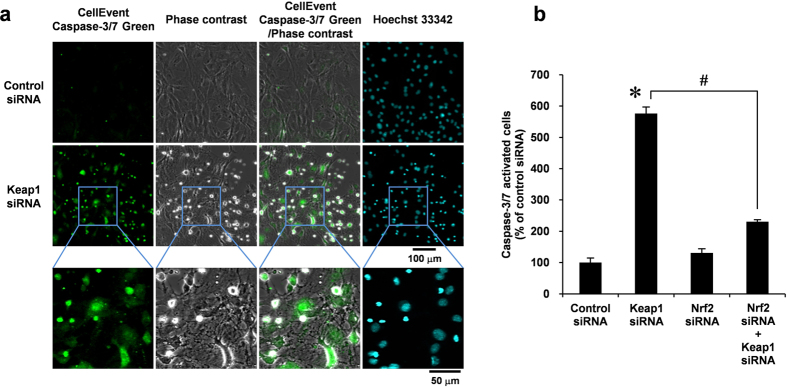
Effect of Nrf2 siRNA co-transfected with Keap1 siRNA in caspase-3/7 activation in VSMCs. (**a**) RASMCs were transiently transfected with Keap1 or control siRNA for 48 h. Growth-arrested RASMCs were stained with CellEvent Caspase-3/7 Green reagent (green) for 90 min. Hoechst 33342 (blue) were added 30 min before the end of the treatment. Fluorescence images were taken by confocal microscopy under fixed exposure conditions. Results are representative of three independent replicates of immunofluorescence images. (**b**) RASMCs were transiently transfected with Nrf2 or control siRNA. After 24 h, the RASMCs were additively transfected with Keap1 or control siRNA for 48 h. Growth-arrested RASMCs were stained with CellEvent Caspase-3/7 Green reagent, and analyzed by flow cytometry. Data are expressed as mean ± SEM of three independent experiments. *P < 0.05 vs. control siRNA. ^#^*P* < 0.05 vs. Keap1 siRNA alone.

To gain insight into the mechanism by which the Nrf2/Keap1 system regulates VSMC apoptosis, we examined whether Nrf2/Keap1 is involved in caspase-3/7 activation using a fluorogenic substrate. In VSMCs, siRNA-mediated depletion of Keap1 increased caspase-3/7 activity (Fig. 5a). Consistent with Fig. 4, flow cytometric analysis revealed that Keap1 knockdown significantly increased the number of caspase-3/7 activated cells, whereas Nrf2/Keap1 double knockdown inhibited caspase-3/7 activation (Fig. 5b), indicating that the overexpression of Nrf2 initiates apoptotic signals in VSMCs.

### Nrf2 regulates vascular cell apoptosis and neointimal expansion

**Figure 6:**
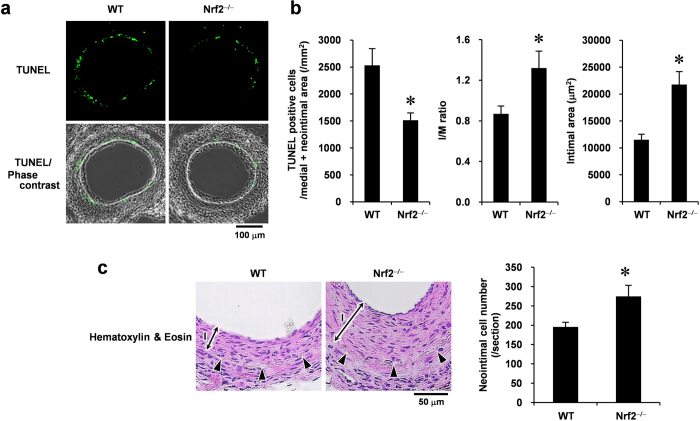
Loss of Nrf2 prevents vascular cell apoptosis and promotes neointimal formation after the vascular injury. (**a**) TUNEL staining and its phase-contrast images of femoral arteries obtained from WT and Nrf2^−/−^ mice at 14 days after injury. (**b**) Quantitative morphometric analysis of TUNEL-positive cells and vessel remodeling in WT and Nrf2^−/−^ mice. I/M ratio indicates intimal area to medial area ratio. Data are expressed as mean ± SEM of three different sections from each of 10 (WT) or nine (Nrf2^−/−^) vessels. (**c**) Hematoxylin & Eosin staining of femoral arteries obtained from the WT and Nrf2^−/−^ mice at 28 days after injury. Arrowheads indicate internal elastic lamina. I, intimal layer. The number of neointimal cells was determined by counting the nucleus. Data are expressed as mean ± SEM of sections from each of five vessels. *P < 0.05 vs. WT mice.

**Figure 7:**
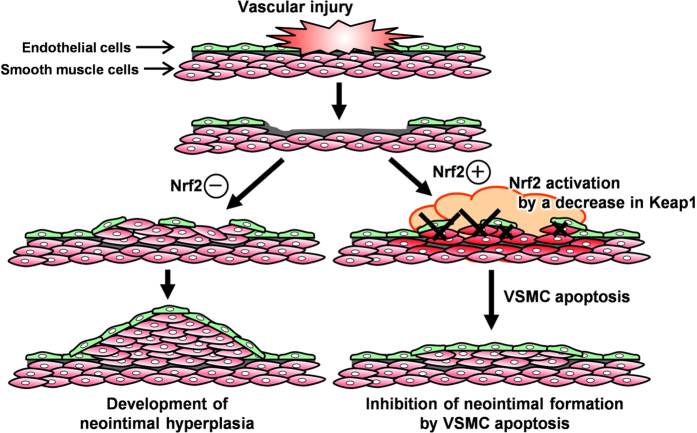
A proposed model for the role of Nrf2/Keap1 system in VSMCs during injury-induced neointimal expansion. The vascular injury induces oxidative stress and a decrease in Keap1 expression, thereby activating Nrf2 in VSMCs. Stress response by Nrf2 activation promotes programmed cell death (“apoptosis”) through activation of caspase-3/7, which prevents excessively neointimal expansion for maintaining vascular function.

To determine the functional significance of the Nrf2/Keap1 system in VSMC apoptosis *in vivo*, we examined the role of Nrf2 in vascular cell apoptosis during the middle stages of neointimal expansion using a mouse femoral artery wire injury model with Nrf2^−/−^ and wild-type (WT) mice. We did not use Keap1^−/−^ mice as this mutation is lethal^19^. Fourteen days after injury, TUNEL analysis revealed that significantly less DNA fragmentations (apoptotic cells) was observed in Nrf2^−/−^ mice than in WT mice (Fig. 6a,b). Quantitative morphometric analysis of the injured vessels revealed that in Nrf2^−/−^ mice the intimal area and intimal/medial (I/M) ratio was increased within 14 days after injury (Fig. 6b). We subsequently examined whether the absence of Nrf2 influences the number of vascular cells in the end stages of neointimal expansion after the vascular injury. The Nrf2^−/−^ mice showed an increase in the number of cells in neointimal lesion at 28 days after wire injury compared with the WT mice (Fig. 6c). These results indicate that Nrf2 influences neointimal formation after injury, at least in part, by inducing VSMC apoptosis that may contribute to vascular remodeling *in vivo* (Fig. 7).

## Discussion

Accumulating evidence has implicated the oxidative stress induced by ROS in the development and progression of many diseases, including vascular occlusive diseases^20^. With improved understanding of the underlying molecular mechanisms, therapeutic interventions that target the oxidative stress response may represent effective strategies for treating various diseases that are driven by prolonged oxidative stress. The Nrf2/Keap1 pathway functions as a biological defense system. The Nrf2/Keap1 system protects against oxidative stress by inducing antioxidant proteins and phase II detoxification enzymes in response to changes in the intracellular redox balance^21^,^22^. Migration and proliferation of VSMC during progression of arteriosclerosis is enhanced by ROS^6^,^23^. In addition, irreversible phenotypic changes in VSMCs can reduce their susceptibility to apoptosis^24^,^25^. We previously reported that the Nrf2 system inhibits PDGF-induced migration of VSMCs by regulating ROS production and elimination^15^. In this study, we further provide the first evidence that the Nrf2/Keap1 system is a key regulator of VSMC apoptosis during vascular remodeling after injury (Fig. 7). These results propose a model in which the Nrf2/Keap1 system plays a dual regulatory role, influencing apoptosis and growth of VSMCs during neointimal formation by inhibiting oxidative stress, thus reducing neointimal hyperplasia.

Generally, the Nrf2/Keap1 system inhibits cell death and carcinogenesis to protect against toxic doses of chemical compounds or diseases that alter the intracellular redox state. This process has been mostly studied with respect to cytotoxicity caused by xenobiotics, anticancer agents, and high concentrations of ROS^26^,^27^,^28^,^29^. However, whether the Nrf2/Keap1 system maintains vascular function by regulating apoptosis under physiological conditions was previously unknown. In this study, we found that Keap1 depletion induces VSMC apoptosis, a process accompanied by increased cellular levels and nuclear accumulation of Nrf2 *in vitro*. Taguchi *et al.*^30^ have reported that liver-specific disruption of Keap1 with the *Atg7* gene in mice induces hepatocellular injury, which is associated with increased Nrf2 levels. Furthermore, caspase-3 activation is involved in cell apoptosis and was observed in VSMCs following Keap1 knockdown. Crucially, the simultaneous depletion of Nrf2 protected these cells against apoptosis. Thus, these results support the hypothesis that Keap1 plays an important role in inducing VSMC apoptosis by controlling Nrf2 activity.

In a mouse model, proliferating cells were identified in the medial and small neointimal layers that were formed on the luminal side of the internal elastic lamina at 1 week after the vascular injury^31^, and the neointimal hyperplasia continued to grow for up to 4 weeks^31^. While VSMC proliferation accelerates during neointimal growth, cell apoptosis also increased in this time^4^. We additionally found apoptotic cells in both the neointimal and medial layers during VSMC proliferative phase after the vascular injury, which is associated with decreased Keap1 and Nrf2 accumulation in αSMA-positive areas. This decrease in Keap1 expression levels is occurred within 7 days (the early stages) after injury. Keap1 degradation is reportedly accelerated under oxidative stress^30^. Our data provide the first evidence that the vascular injury also decreases Keap1 mRNA level, suggesting that the amount of Keap1 is regulated by gene expression and protein degradation. Furthermore, in Nrf2^−/−^ mice, VSMC apoptosis was decreased in the middle stages of neointimal expansion. These observations are consistent with *in vitro* data and suggest that oxidative stress, which was induced by the vascular injury, reduced the function of Keap1, thus causing VSMC apoptosis. In contrast, a previous study revealed that adenovirus-mediated Nrf2 overexpression reduces the VSMC apoptosis in the rabbit aortic balloon denudation model^32^. Therefore, Nrf2 overexpression by adenovirus infection may increase the possibility of affecting the physiological role of Nrf2 by infection-induced excessive oxidative stress and Nrf2 overexpression without Keap1 co-transfection. Altogether, these results suggest that the coordinated functional regulation of Nrf2 by Keap1 is important for the vasculature homeostasis.

In this study, we observed that the intimal layer and I/M ratio of the injured vessels of Nrf2^−/−^ mice was greater than that of WT mice at 14 days after the injury. In addition, the Nrf2^−/−^ mice showed a significant increase in the number of vascular cells in neointima at the end of neointimal formation (28 days) after injury compared with the WT mice. Consistent with these results, we previously demonstrated that Nrf2^−/−^ mice experience markedly increased neointimal expansion during the late stages of vascular wound repair when the compared cell density does not differ from that in the WT mice^15^. Neointimal growth is influenced by the balance between VSMC proliferation and apoptosis, which determines the number of VSMCs in the arterial wall after injury^33^. These observations indicate that Nrf2 is involved in an increase in the number of VSMCs in the neointimal layer and suggest that reduced VSMC apoptosis during the proliferative phase causes an increase in the final number of VSMCs, thereby exacerbating intimal thickening in the Nrf2^−/−^ mice. Thus, the Nrf2/Keap1 system-induced VSMC apoptosis during neointimal formation benefits luminal patency under physiological conditions.

In this study, we confirmed the induction of Nqo1 and Hmox1 by Keap1 depletion as exemplary Nrf2 target genes in VSMCs. It has been previously reported that Nrf2 target gene Hmox1 and its metabolic product, bilirubin, inhibit VSMC growth and proliferation in addition to migration^34^,^35^ and that Nqo1 also suppresses VSMC proliferation^16^. Therefore, VSMC apoptosis is also mediated by Nrf2 target genes. The Nrf2/Keap1 is established as a cytoprotective system to protect against oxidative damage by altering the expression of target genes^13^,^14^; however, Nrf2 also plays a role in the transcription regulation of drug-metabolizing enzymes, such as cytochromes P450, which produce ROS in metabolic processes, as well as antioxidant proteins^36^,^37^. These reports suggest that the Nrf2/Keap1 system has a broad range of exquisite biological functions. Thus, the Nrf2/Keap1 system may function to remove unnecessary cells from the tissues for maintaining homeostasis. However, because Nrf2 affects the expression of over 200 target genes^20^, suggesting the formation of functional networks, the precise target genes involved in the induction of VSMC apoptosis has not been clarified. Our preliminary data showed that the loss of Nrf2 prevents vascular injury-induced Hmox1 gene expression (unpublished observations). Therefore, further studies are required to elucidate the functional roles of the Nrf2 target genes in blood vessels.

In conclusion, this study is the first to characterize the critical importance of the Nrf2/Keap1 system in the induction of VSMC apoptosis during neointimal formation. We report that the Nrf2/Keap1 system maintains the functional integrity of blood vessels after injury. Nrf2 activation improved the anti-oxidative capacity and contributed to protection against oxidative damage^13^,^14^. Many studies have reported the pathological significance of Nrf2 in protection against oxidative stress. For example, Nrf2^−/−^ mice are more susceptible to allergen-induced asthma^38^, septic shock^39^, and lupus-like autoimmune nephritis^40^. Clinically, dimethyl fumarate, an Nrf2 inducer, has already received the US Food and Drug Administration approval for the treatment of relapsing-remitting multiple sclerosis^13^. Furthermore, another promising Nrf2 inducer is 2-cyano-3,12-dioxooleana-1,9(11)-diene-28-oic acid-methyl ester, which is a candidate for treating pulmonary hypertension and for chronic kidney disease associated with type 2 diabetes^13^. Thus, the development of therapies targeting the Nrf2/Keap1 system is anticipated. Whereas Nrf2^−/−^ mice grow normally under normal conditions, Keap1^−/−^ mice died postnatally because of hyperkeratosis in the esophagus and forestomach. These fatalities can be overcome by breeding with Nrf2^−/−^ mice^19^. Thus, it is likely that systemic forced activation of Nrf2 causes negative health outcomes. These results indicate that appropriate activation of Nrf2 under the control of Keap1 in the target tissues is important for homeostasis but not for continuous hyperactivation of Nrf2. Our findings provide insight into the Nrf2/Keap1 system and recommend it as a potential therapeutic target to reduce the effects of postangioplasty restenosis and atherosclerosis, which are associated with oxidative stress.

## Materials and Methods

### Materials

Antibodies to Nrf2 (sc-722) and Lamin B1 (sc-20682) were acquired from Santa Cruz Biotechnology (Santa Cruz, CA). Antibodies to Keap1 (#8047) and cleaved caspase-3 (Asp175) (#9661) were acquired from Cell Signaling Technology (Beverly, MA). Anti-αSMA antibody (#03-61001) was from American Research Products (Belmont, MA). Anti-actin antibody (A2066) was acquired from Sigma-Aldrich (St. Louis, MO). Proteinase K was acquired from Qiagen (Hilden, Germany). ProteoGuard EDTA-Free Protease Inhibitor Cocktail was acquired from Takara Bio (Shiga, Japan). All other reagents that were used were of the highest grade commercially available.

### Animals

All animal experiments were conducted under the control of the Committee Regulation of Animal Care and Welfare of Showa University. Male C57BL/6 mice (8 weeks old) were purchased from Japan SLC (Shizuoka, Japan). The Nrf2^−/−^ mice were established by Itoh *et al.*^41^. C57BL/6 mice were mated with Nrf2^−/−^ mice. WT and deficient mice progenies were selected by their respective genotypes, and the lines for WT or deficient mice were established. All studies were carried out in accordance with protocols approved by the Institutional Animal Care and Use Committees of Showa University, in accordance with the Standards Relating to the Care and Management of Experimental Animals in Japan.

### Cell culture

RASMCs were isolated from male Sprague Dawley rat thoracic aortas by enzymatic digestion, as developed by Travo *et al.*^42^. Cells were grown in Dulbecco’s modified Eagle’s medium that was supplemented with 10% bovine serum and was used at between passage 8 and 13. To exclude the effect of serum on cell proliferation, cells were starved for 24 h with serum-free media before experiments.

### siRNA transfection

Predesigned siRNAs were obtained from Applied Biosystems. VSMCs were seeded into culture dishes 1 day prior to transfection. Transfection of siRNA (5 nM) was performed using Lipofectamine RNAiMAX reagent (Invitrogen) according to the manufacturer’s protocol.

### Vascular injury

Transluminal mechanical injury of the femoral artery was directly induced in the neointimal layer using a method developed by Sata *et al.*^31^. Briefly, the animals were anesthetized with sodium pentobarbital (65 mg/kg, i.p.), and the left femoral artery was exposed by blunted dissection. More than 5 mm of a straight spring wire (0.38 mm in diameter, No. C-SF-15-15, Cook Medical, Bloomington, IN) was carefully inserted into the femoral artery toward the iliac artery from the exposed muscular branch artery. The wire was left in place for 1 min to denude and dilate the artery and then removed, and the proximal portion of the muscular branch artery was secured by stitching with silk suture loops. The flow of the femoral artery remained and dilated the injured artery. At 14 days after injury, arteries were excised using two methods of vessel harvesting. For vessels intended for immunohistochemistry or immunofluorescence staining, mice were anesthetized and perfused with 4% paraformaldehyde (PFA) for tissue fixation. Excised arteries were fixed in 4% PFA overnight, embedded in paraffin, and mounted on slides (5-μm-thick sections). For real-time polymerase chain reaction (PCR) or immunoblotting, mice were anesthetized and perfused with RNAlater solution (for real-time PCR) or PBS (for immunoblotting). After removing the adhering fat tissue, excised whole-arteries were quickly frozen in liquid nitrogen and stored at −80 °C until RNA extraction or protein preparation. The right femoral arteries were simultaneously excised with the left femoral arteries as control vessels.

### TUNEL assay and immunofluorescent staining

DNA fragments generated in response to apoptotic signals were detected by TUNEL analysis using *in situ* Cell Death Detection Kit (Roche Diagnostics, Basel, Switzerland) according to the manufacturer’s instructions. For vessels, paraffin-embedded tissue sections were deparaffinized, permeabilized with 20 μg/ml proteinase K in 10 mM Tris-HCl (pH 7.6) at room temperature for 15 min, and blocked with 10% normal goat serum in PBS for 1 h. After TUNEL assay, sections were stained with anti-Nrf2 antibody (1:200 dilution) or anti-αSMA antibody (1:500 dilution) at 4 °C for 18 h, followed by an Alexa Fluor 546 goat anti-rabbit IgG or an Alexa Fluor 546 goat anti-mouse IgG at room temperature for 1 h. Sections were mounted with Vectashield (Vector Laboratories, Burlingame, CA) and observed with Fluoview FV10i confocal microscope (Olympus, Tokyo, Japan) using the 405-, 473-, and 559-nm diode lasers. Quantitative morphometry of three different sections from each of 10 (WT) or 9 (Nrf2^−/−^) arteries at 300-μm intervals were analyzed using the FV10-ASW 3.0 software (Olympus). For VSMCs, the cells were quickly rinsed in ice-cold PBS, fixed in freshly prepared 4% PFA in PBS at room temperature for 30 min, and permeabilized in ice-cold 0.1% Triton X-100 in 0.1% sodium citrate for 2 min. After the TUNEL assay, cells were observed with a Biozero BZ-8000 fluorescence microscope (Keyence, Osaka, Japan). DAPI was used to visualize the nucleus. Controls with no primary antibody and/or no terminal deoxynucleotidyl transferase demonstrated no fluorescence labeling.

### Immunohistochemical analysis

Paraffin-embedded tissue sections were sequentially deparaffinized and rehydrated in xylene, 100% ethanol, 90% ethanol, 70% ethanol, and distilled water. Deparaffinized sections were autoclaved in 10 mM sodium citrate buffer (pH 6.0) for antigen retrieval (15 min at 121 °C), and incubated in 3% hydrogen peroxide in methanol (10 min at room temperature) to quench endogenous peroxidase activity. After incubation for 1 h in blocking buffer (10% normal goat serum in PBS), sections were stained with anti-cleaved caspase-3 (Asp175) antibody (1:200 dilution), followed by a peroxidase- and a second antibody-conjugated amino acid polymers (Nichirei Biosciences, Tokyo, Japan). Staining was developed using a DAB Kit (Vector Laboratories). Sections were counterstained with hematoxylin. For counting the number of neointimal cells, deparaffinized sections were stained with hematoxylin and eosin and were subsequently analyzed using the ImageJ 1.48j software (NIH).

### Quantitative real-time PCR

Total RNA was isolated from VSMCs or femoral arteries using RNeasy Mini Kit (Qiagen), and first-stranded cDNA was synthesized with PrimeScript RT Master Mix (Takara Bio). The duplex TaqMan real-time PCR using a FAM-labeled probe for the target mRNA and a VIC-labeled probe for the housekeeping gene were performed according to the manufacturer’s protocol using the StepOne real-time PCR system (Applied Biosystems) and TaqMan Fast Advanced Master Mix (Applied Biosystems). The mRNA levels were measured as the relative ratio to glyceraldehyde-3-phosphate dehydrogenase mRNA (*in vitro*) or β-actin mRNA (*in vivo*). All predesigned PCR primers and TaqMan MGB probes were purchased from Applied Biosystems. The assay ID of the probes used in this study were as follows: rat Nrf2, Rn00477784_m1; rat Nqo1, Rn00566528_m1; rat Hmox1, Rn01536933_m1; rat Keap1, Rn00589292_m1; and mouse Keap1, Mm00497268_m1.

### Preparation of nuclear and cytoplasmic extracts

VSMCs were harvested by trypsinization, washed with PBS, and pelleted by centrifugation. Nuclear and cytoplasmic extracts were prepared from the cells using NE-PER Nuclear and Cytoplasmic Extraction Reagents (Thermo Scientific) according to the manufacturer’s instructions. The protein levels were measured as the relative ratio to Lamin B1, a marker of nuclear fractions, or actin.

### Immunoblotting

VSMCs were washed in ice-cold PBS and solubilized with 4% sodium dodecyl sulfate. Femoral arteries were homogenized with lysis buffer (20 mM Tris-HCl, pH 7.4, containing 1% Nonidet P-40, 150 mM NaCl, and protease inhibitor cocktail). The homogenates were centrifuged at 13,000 ×g for 15 min, and the resulting supernatants were used for immunoblot analysis. Protein concentration was determined using Pierce BCA Protein Assay Kit (Thermo Scientific) according to the manufacturer’s instructions. Protein samples obtained from vessels or cells were separated using sodium dodecyl sulfate-polyacrylamide gel electrophoresis and transferred to polyvinylidene difluoride membranes. Membranes were blocked with TBS containing 0.2% Tropix I-block (Applied Biosystems) and 0.2% Tween 20 and were incubated overnight at 4 °C with primary antibodies. After incubating with secondary antibodies, proteins were detected by ECL (GE Healthcare, Little Chalfont, United Kingdom) chemiluminescence. Molecular weight was calculated with pre-stained protein marker that was applied to the same gel run samples. The relative densities were analyzed using ImageQuant TL software (GE Healthcare).

### Identification of apoptotic cells by Hoechst 33342 staining

VSMCs were stained with Hoechst 33342 (2 μg/ml) for 30 min, washed with PBS, fixed in freshly prepared 4% PFA in PBS for 10 min at room temperature, and rinsed with PBS. Cells were observed with a Biozero BZ-8000 fluorescence phase-contrast microscope with digital camera output (Keyence). The percentage of apoptotic cells were determined by counting shrunken cells in three random fields using ImageJ 1.44p software (NIH).

### Apoptosis assay with FITC-labeled annexin V and PI staining

VSMC apoptosis was measured by annexin V and PI staining using the annexin V Apoptosis Detection Kit FITC (eBioscience, San Diego, CA) according to the manufacturer’s protocol. Briefly, siRNA transfected-VSMCs were trypsinized, washed with PBS and annexin V-binding buffer, and re-suspended in the binding buffer containing annexin V and PI. After incubating at room temperature in the dark for 30 min, the cells were filtered using a 35-μm filter and were immediately analyzed using the Epics XL flow cytometer (Beckman Coulter, Brea, CA). The apoptotic cells were defined as annexin V positive and PI negative cells (red square at right lower quadrants), whereas live cells remained unstained.

### Caspase-3/7 activation

Caspase-3/7 activation was detected using CellEvent Caspase-3/7 Green reagent (Molecular Probes) according to the manufacturer’s protocol. For confocal immunofluorescence microscopy, CellEvent Caspase-3/7 Green reagent was added to VSMCs in glass-based dishes at 37 °C for 1 h, washed with ice-cold PBS, and fixed in freshly prepared 4% PFA in PBS at room temperature for 10 min. The cells were observed with Fluoview FV10i confocal microscope (Olympus) using the 473-nm diode laser. For flow cytometry, VSMCs in 6-well plate were added CellEvent Caspase-3/7 Green reagent. After incubating at 37 °C for 1 h, the cells were trypsinized, washed with PBS, filtered using a 35-μm filter, and immediately analyzed using the Epics XL flow cytometer (Beckman Coulter). The excitation/emission maxima for the CellEvent Caspase-3/7 Green reagent are 502 nm/530 nm.

### Statistical analysis

All values are expressed as means ± SEM. The significance of the differences between the two groups was evaluated by Mann–Whitney’s U test. The values in more than three groups were tested by Kruskal–Wallis test and were followed by Scheffe test. Statistical significance was accepted at *P* < 0.05.

## Additional Information

**How to cite this article**: Ashino, T. *et al.* Nrf2/Keap1 system regulates vascular smooth muscle cell apoptosis for vascular homeostasis: role in neointimal formation after vascular injury. *Sci. Rep.* **6**, 26291; doi: 10.1038/srep26291 (2016).
